# News and Miscellany

**Published:** 1903-09

**Authors:** 


### News and Miscellany.

Dr. J. Shelton Horsley, late of El Paso, Texas, has been called to the Chair of Clinical Surgery in the Virginia Medical College, Richmond, Va.

---

The Lee County Medical Society was organized at Giddings, Texas, September 3rd, inst., with Dr. A. C. Connor, of Lexington, President; Dr. S. G. Northrop, of Giddings, as Secretary.

---

Dr. I. P. Sessions, of Rockdale, is in the service of the Public Health Department of Texas, and under orders from State Health Officer Tabor is on inspection service in Monterey, Mexico.

Wanted to Buy.—A practice for cash, or will exchange property. Location must be in the black land section, and income not less than $2000 a year. Quick. Address Mac., care of the editor of the Texas Medical Journal.

---

For Sale; a Bargain.—A neatly articulated skeleton; cost $50, offered for $25 f. o. b. at Liberty Hill, Texas; also a set of DePuy's adjustable fiber splints, 45 pieces, cost $15, offered for $7.50. J. W. Thorpe, Liberty Hill, Texas.

---

A provoking error occurred in Dr. Allen J. Smith's article in the August number, "Experiments to Determine the Disinfecting Value of the Germicide Gas Generator." On page 45, tenth line, for "each cubic foot of air space" read "for each 1000 cubic feet of air space."

---

Preceptors and students will do well to correspond with Dr. J. M. Bodine, Dean of the Medical Department, University of Louisville, before deciding upon a medical college. The Louisville Medical College is second to the oldest, and is one of the best in the South. Please see announcement in our advertising pages.

---

The yellow fever is spreading in Mexico, and at this writing is said to have reached Nuevo Laredo, Mexico, just across the river from Laredo, Texas. A panic has struck that town, according to the press dispatches, and people are flying to the interior of the State for safety. A bomb-proof quarantine meantime is maintained on the Rio Grande by the Texas authorities and the Marine Hospital Service of the United States—all of which seems useless and absurd *IF* a mosquito is the *only* means of spreading the disease, as held by the Marine Hospital Service of the United States et al. You can't quarantine a mosquito. Meantime fumigation with sulphur and formaldehyde goes on of all baggage just the same as if it was still feared that *maybe* fomites *can* carry the disease after all. It is paradoxical. The mosquito doubtless communicates the disease, but nothing on earth will convince me that it may not be also spread by fomites (clothing, goods, etc.) packed in boxes or trunks. I've seen it too often. Dr. Tabor is right. He will not risk it. The people would not hold him guiltless if he did.

---

Something Worth Having.—M. J. Breitenbach Co., the importers of Gude's Pepto-Mangan, have issued a beautiful colored lithograph map, showing, greatly magnified, most of the pathogenic bacteria. It is invaluable as a diagnostic aid in studying these germs under the microscope. It is a chart, to be hung in the office or laboratory. A copy will be sent free for the asking if the "Red-Back" be mentioned. Of this work the (N. Y.) *Medical News* (July 18th) says, editorially: "No text-book and no one work on pathogenic bacteria contains such a number of excellent diagnostic illustrations, nor such beautiful examples of lithographic art, as these. Many physicians are too far from libraries and laboratories to be able to put into practice the training of their college days. They need just such a set of reference plates to be able to make microscopical examinations. The recognition of this need and the care that has been taken to fill it shows a spirit of enterprise in this firm that we wish might serve as an example to others. For, if, instead of advertising to the public, the manufacturers of drugs would make such valuable contributions to science as lies in their power, there might be more sympathy between them and physicians. The full set of sixty cuts has been prepared to send to any physician who writes for them, from the firm of M. J. Breitenbach Co., New York."

### Specimen Letters to the "Red-Back."

MARFA, TEXAS, September 12, 1903.
*Editor Texas Medical Journal.*

DEAR DOCTOR: I take pleasure in renewing my subscription for the "Red-Back." It is a wide-awake journal and brings something interesting every month.

Yours, with best wishes,
FRANCIS D. COLTRIN.

GENTRY, TENN., September 11, 1903.
*Editor Texas Medical Journal.*

Enclosed find check for $2.00 for subscription for years 1903 and 1904 to February, 1905. I can not do without the "Red-Back."

Respectfully,
W. S. FARMER, M. D.

BECKVILLE, TEXAS, September 16, 1903.
*Editor Texas Medical Journal.*

DEAR DOCTOR: Enclosed please find money order for $1.00 as payment for yours, the journal of all journals. Just please continue to let it come, and make it as hot for quacks in the future as you have done in the past. I hope to be able to meet you at our next State meeting.

With best wishes, I am

Yours fraternally,
R. F. HARNESBERGER.

RARE CHANCE FOR DOCTOR.—Residence and $3000 practice; railroad town, county seat. Little opposition. Also drug store and stock of drugs. Address "West Texas," care TEXAS MEDICAL JOURNAL.

For Sale.—In small town, four room cottage, wind mill and outbuildings; office on separate lot; $3000 practice thrown in; collection 90 per cent. No opposition. Price $600. U. E. G. Dyer, M. D., Star, Texas.

---

The Southwestern Tri-State Medical Society (Texas, Oklahoma and Indian Territory) will hold its fourth annual meeting in Dallas, Texas, September 29th and 30th. Dr. J. C. Loggins, President; Dr. C. M. Rosser, Secretary.

---

For Sale.—Well established practice of from $1500 to $2000. Five-room dwelling house, barns and all necessary outbuildings, one and one-half acres of land, in small inland town. No competition. Price, $600; terms, $350 cash, $250 November 1, 1904. Will give possession November 1, 1903. Address, Dr. B., Overbrook, I. T.

---

The directors of the Memphis Medical College have established a lectureship, named in memory of the late William E. Rogers, M. D., founder of that institution. The lectureship was established upon the suggestion of the faculty. Once during each session a lecture will be given by a specialist in some branch of medical science, at the invitation of the faculty. The first lecture will be given about the middle of November by Dr. Charles H. Hughes, of St. Louis.

---

Death of the Venerable Medical Director of all the Confederate States Hospitals.—Dr. Samuel Hollingsworth Stout, known all over the world as the head of the Confederate States Hospital Department during the War of Secession, died at his home in Clarendon, Texas, September 18th, inst., at the age of 82 years. Dr. Stout removed to Clarendon from Dallas only a few months ago. This sad news comes after this issue was on press, and I have room only for this brief comment. In next issue there will be a complete biographical sketch of the illustrious physician and educator, with a portrait from his latest photograph.

---

The State Board of Medical Examiners will meet at Waco, October 13th (prox.), and sit four days.

